# Experimental Analysis of the Stress State of a Prestressed Cylindrical Shell with Various Structural Parameters

**DOI:** 10.3390/ma15144996

**Published:** 2022-07-18

**Authors:** Nurlan Zhangabay, Bayan Sapargaliyeva, Akmaral Utelbayeva, Alexandr Kolesnikov, Zhumadilla Aldiyarov, Serik Dossybekov, Esenbek Esimov, Bolat Duissenbekov, Roman Fediuk, Nikolai Ivanovich Vatin, Myrzabek Yermakhanov, Saule Mussayeva

**Affiliations:** 1 Department of Construction and Construction Materials, M. Auezov South Kazakhstan University Shymkent, Shymkent 160012, Kazakhstan; nurlan.zhanabay777@mail.ru (N.Z.); ermurat1234@mail.ru (Z.A.); bakdulet777@mail.ru (S.D.); e.esimov777@mail.ru (E.E.); maraltai@mail.ru (B.D.); m.yermakhanov1968@mail.ru (M.Y.); s.musaeva1971@mail.ru (S.M.); 2 Abai Kazakh National Pedagogical University, Almaty 050010, Kazakhstan; bonya_sh@mail.ru; 3 Polytechnic Institute, Far Eastern Federal University, 690922 Vladivostok, Russia; 4 Peter the Great St. Petersburg Polytechnic University, 195251 St. Petersburg, Russia; vatin@mail.ru

**Keywords:** steel shell, prestressing, steel wrapping, structural parameters, stress state, strength

## Abstract

The paper presents the results of experimental studies of the features of the operation of prestressed shells, taking into account the various structural parameters of the prestress. It is established that when the winding angle changes from perpendicular to the shell axis to 75° and 65°, the circumferential stresses decrease 1.4 times and 1.2 times, respectively, and the axial stresses increase five and three times, which are two and four times lower than the circumferential, from which it can be concluded that the reduction in the winding angle to the longitudinal the axis of the shell has a positive effect on the stress state of the structure. The study also found that with an increase in the diameter of the winding wire from 1 to 2 mm and a change in the winding angle, the same nature of the stress distribution is observed, but the values of the stress state parameter change, so the efficiency increases up to 25% due to an increase in the winding thickness, depending on the pitch, angle and thickness of the winding, which favorably affects the strength and the bearing capacity of the structure as a whole by increasing the value of the stress state parameter. Thus, the results of the analysis will allow us to use in more detail the possibility of controlling the stress–strain state of the prestressed shell by changing the design parameters, and the results obtained can be used in design or construction, as well as when increasing the strength characteristics of the structure, which allows us to create a high-tech design optimal for these operating conditions, which can positively complement the studies conducted earlier in this direction.

## 1. Introduction

The creation of technical potential to ensure sustainable development of the economy is directly related to the introduction of sustainable, cost-effective technologies, and design solutions in strategically oriented areas oil and gas production, oil refining, petrochemistry, energy and transport.

Achieving sustainable development largely depends on the creation of highly economical, reliable and cost-effective technologies, design and structural solutions.

The high rates of development of oil, gas, as well as chemical and energy industries require a powerful intensification of construction of shell structures, which include pipelines, reservoirs, gasholders, vessels and pressure vessels. Significant funds are also required to restore the operated cylindrical shells’ bearing capacity.

Such structures’ operation, construction, and design involve high material costs and environmental pollution risk [1,2,3,4,5]; therefore, these structures refer to especially critical structures, and their development should be based on strictly substantiated scientific, technically possible, fundamentally new structural and economically viable solutions.

One of the ways of such solutions, the most recognized, is the use of the prestressing method, which is created by winding high-strength wire or tape on the shell body [6].

At the same time, prestressing can be used to increase or restore the cylindrical shell’s bearing capacity as well as to eliminate the thin-walled structure walls’ deformation under operational loads [7,8].

One of the important advantages of such structures is the ability to control the structure’s stress state by selecting the prestressing structural parameters: force, pitch and winding angle of the wrapping thread.

The relevance of research on this issue lies in the fact that the world demand for raw materials, such as oil and gas, increases the need for production; therefore, the question of transportation and storage arises, where an increase in oil exports also predetermined an increase in the volume of pipeline transport construction. The transportation of oil and oil products to the places of consumption is largely complicated by the distant location of deposits from industrial centers. Hence, shells of exceptionally high capacity and reliability should be created, which is possible only on the basis of fundamentally new design solutions.

Shell steel structures have been widely used for the storage and transportation of liquids for a long time. There is a large amount of research in the field of the static and dynamic strength of such structures. The need to operate such structures for oil storage and transportation does not exclude the occurrence of accidents or unforeseen technical shutdowns as a result of problems caused by malfunctions during operation, aging of the structure and various deformations, which demonstrated the need for additional studies of the strength of steel shells and the implementation of new engineering solutions. The works [9,10,11,12,13,14,15,16,17,18,19,20,21,22,23,24] present the results of studies, including the modeling of shell structures under various static and dynamic loads, where studies were also carried out taking into account the deformations of the shell body (pipelines, reservoirs, cisterns, etc.). In the studies [25,26,27,28,29,30,31,32,33,34,35,36,37,38], work was carried out to study the effect of damage and defects in steel wrappings on the stiffness and natural frequencies of prestressed pipes as well as the characteristics of metals. Nonlinear analysis of prestressed round cylindrical shells’ free vibrations was studied, and the simultaneous influence of the prestressed state and the elastic foundation on the eigenfrequencies of shells under various boundary conditions was considered in detail. The initial tension force was considered as the prestressing parameter. Recommendations on the design, selection of pipes and construction of underground and aboveground pipelines were considered. Pipes made of cast iron, ductile iron, steel, plastic polyethylene, fiberglass, concrete, fiber and asbestos–cement pipes were considered. With reference to relevant standards, several sections are devoted to the structural design of rigid, semi-rigid and flexible pipes, longitudinal stresses, steel pipe wall thickness, resistance to internal forces, pipe restraint and thrust blocks. The risks that lead to losses were considered, which is important to understand which threats increase the likelihood of risk in the pipeline system and how to evaluate them.

The results of the above works [9,10,11,12,13,14,15,16,17,18,19,20,21,22,23,24,25,26,27,28,29,30,31,32,33,34,35,36,37,38] show the general issues of construction and design of steel structures with and without prestressing shells.

However, these methods do not allow completely assessing the effect of winding on shell structures, whereby selecting the above structural parameters and studying their effect on the stress state of prestressed shell structures, it is possible to positively supplement the studies carried out earlier.

In this connection, the significance of the study on the use of prestressing, taking into account the influence of structural parameters on the stress state of shell steel structures, lies in a new approach to improving strength characteristics and saving metal by selecting the necessary design parameters for newly designed and existing steel structures. It should be noted that in this direction, there are practically no experimental studies on the influence of prestress parameters (pitch, tension force, winding angle and winding thickness) on the stress–strain state of the shell wall, which gives great importance to the study. In view of the above, the research purpose is an experimental analysis of the effect of prestressing parameters on the stress state of a prestressed cylindrical shell.

## 2. Materials and Methods

The modeling of geometric dimensions and design parameters was made based on the correspondence of simple mechanical and affine similarity between the model and the full-scale design. This theory is based on the dimensions of physical quantities. In this case, the modeling scales’ constancy was established between the model and nature parameters. For thin-walled steel shell structures, in accordance with the tasks of experimental studies, the scaling of models of samples of two types was chosen: direct and affine to the natural shell size. Experimental studies were divided into stages, sub-stages and series regarding the method of modeling (Table 1).

**Figure 1:**
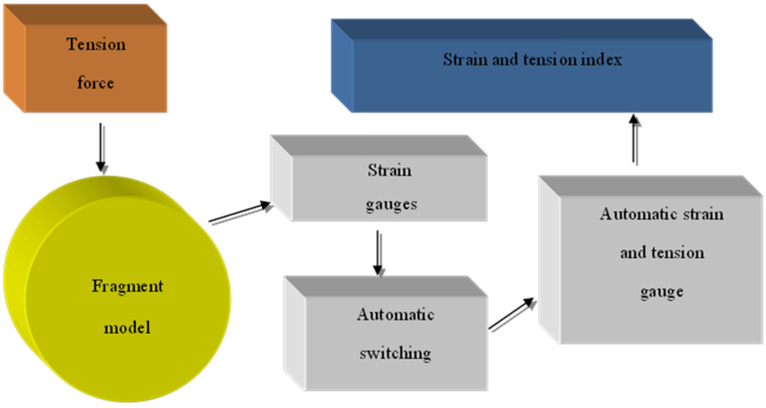
Flowchart of experimental stand.

The tests were carried out on cylindrical shell fragments of different diameters, pitch, winding angle and winding thickness according to Table 1, where the shell width was taken as 200–300 mm. Experimental studies were made on the stand (Figure 1).

When testing the cylindrical shell fragments, the following geometric dimensions and prestressing parameters were modeled:

- Diameter and thickness of the cylindrical shell;
- Pitch, angle and winding force of the wrapping thread, diameter of the wrapping wire.

The prestressing load was simulated by winding a high-strength wire with a diameter of 1.0 and 2.0 mm onto the cylindrical shell fragment with a tension force, different pitch and winding angle of the thread.

During testing the shell fragments, the stresses in the shell wall were determined at characteristic points in the circumferential and axial directions.

Single-element paper-based strain gauges with bases of 5 and 10 mm were used as primary transducers for measuring strains. The resistance of the sensor ranges from 50 to 100 ohms. Calibration was carried out in a standard way.

**Figure 2:**
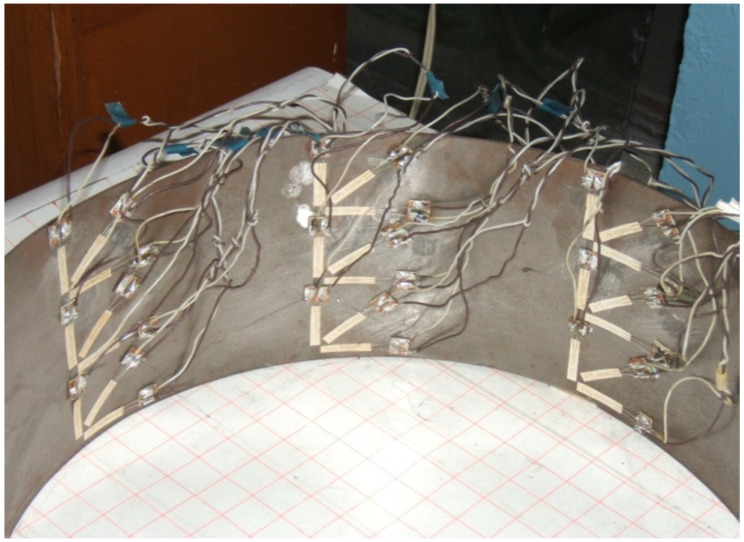
A fragment of the scheme placement of strain gauges on a paper basis with a base of 5 and 10 mm around the circumference of the shell.

The placement was carried out at eight points along the shell fragment generatrix. The strain gauges were glued in such a way that at one point of the wall, it was possible to measure the circumferential, axial and radial stresses in the shell wall in accordance with Figure 2. In total, when testing one sample, measurements were taken from 24 active strain gauges.

In addition, the choice of measuring apparatus, equipment and instruments was carried out, taking into account the tasks assigned to experimental studies.

**Figure 3:**
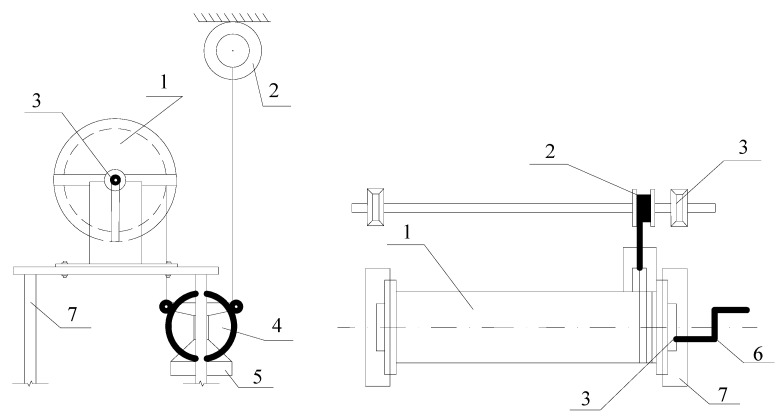
Special installation for winding the steel wrapping on the shell body. 1—Shell fragment; 2—Coil with wire; 3—Coil; 4—Block; 5—Container for load; 6—Handle; 7—Frame.

The winding of the steel wrapping on the shell wall was carried out by a special installation (Figure 3), which allows controlling the wire tension force through a system of dynamometers as well as the winding pitch of the wrapping thread. The winding force of the wrapping thread for all test series was taken equal to 0.75 of the wire breaking force, which was taken according to GOST 5663-79 [19].

The shell fragments were made of steel sheets 0.5 mm, 0.6 mm and 0.7 mm thick, according to GOST 16523-70 [20]. The general view of the shell fragment prepared for testing is shown in accordance with Figure 2. The shell model material is St 8 steel, and that for the wrapping is St 15 according to GOST 27069-86 [21].

The winding force for the prestressed shell fragment was also taken to be less than the critical one and was taken equal to 0.75 *S_critical_* (*S_cr_*), where *S_cr_* is the wire tension force at which the shell buckles, which was determined by the formula [22]:

(1) $$S_{cr}=\frac{2}{r}\sqrt{E_{1}JE_{2}\delta_{2}}$$

where *E*_1_, *E*_2_—the elastic moduli of the shell and wrapping materials; *E*_1_*J*—the ring stiffness; *J*—moment of inertia determined by (2); *δ*_1_, *δ*_2_—the shell and wrapping thicknesses, *μ*—0.3 (Poisson’s ratio).

(2) $$I=\frac{\delta_{c}^{3}}{12(1-\mu^{2})}$$

Thus, these parameters simulate the main possible options for conducting experimental studies, since the thread tension force is 0.75 of the critical one, which gives sufficient force for conducting the study, excluding the winding break. It is determined by Equation (1), taking into account the geometric and mechanical parameters of the model, which will give maximum accuracy in the experimental study and is accepted as one of the parameters of the prestress.

Based on the foregoing, the choice of a methodology for carrying out experimental studies is based on a set of rules, which consists of reproducing the technological and structural parameters as close as possible to real conditions in accordance with the purpose of experimental tests.

## 3. Results and Discussion

To assess the influence of the winding angle of the wrapping thread on the stressed state of the prestressed shell, tests were made on the cylindrical shell fragments of various diameters and wall thicknesses at angles of 65°, 75°, and 90° to the longitudinal shell axis and the winding pitch of the wrapping thread equal to d, 3d and 5d, where d is the wrapping diameter (Table 1).

The selection of such winding angles and pitch is based on the technical possibility of winding the wrapping thread on the shell without slipping. The preliminary compression force value of the shell wall fragment was taken equal to *S_cr_* (1). The wrapping options with wire diameters of 1.0 mm and 2.0 mm were considered, where the critical force of the steel wire was also taken to be 0.75 from the wire rupture according to [19].

**At the first stage** of testing, the diameter of the tensioned wire was assumed to be 1.0 mm at the winding steps a = d, a = 3d, a = 5d. At the same time, different wall thickness and shell diameter were considered. In general, the results of experimental studies are shown in Table 2.

**Figure 4:**
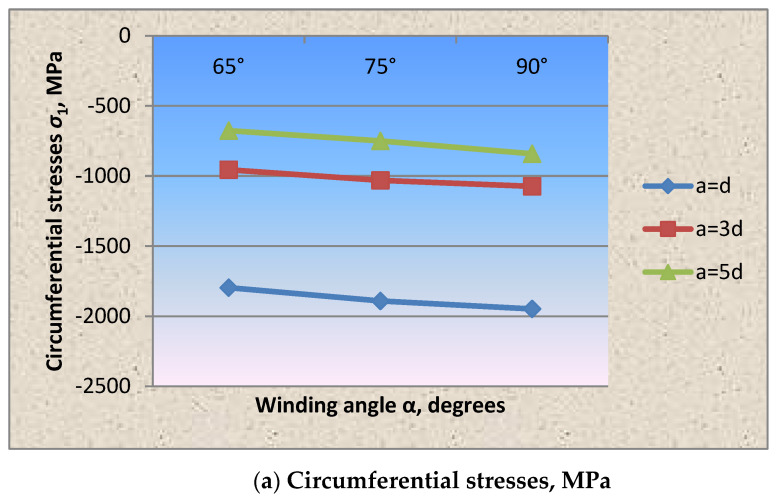

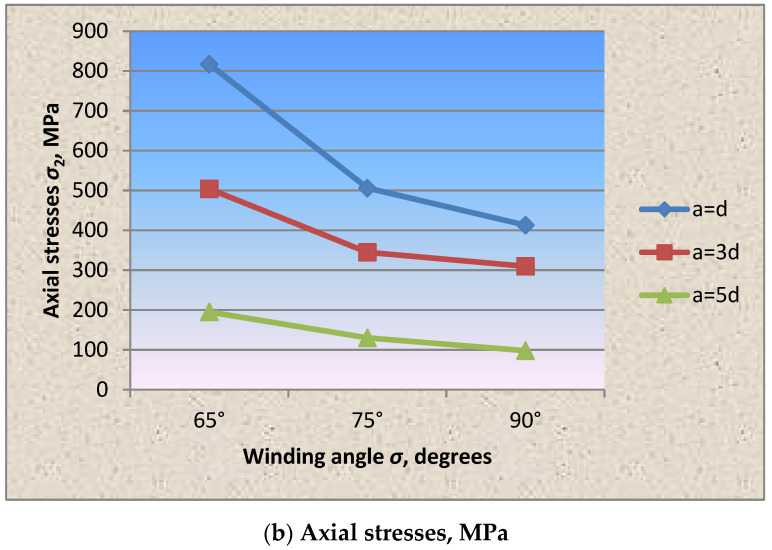
Dependence of the circumferential (**a**) and axial (**b**) stresses in the shell wall fragment with the winding wire diameter of 1.0 mm.

The analysis of the circumferential and axial stresses’ distribution in the shell wall fragment shows that the greatest circumferential compressive stresses in the shell wall due to prestressing occurring when the winding is perpendicular to the longitudinal axis, which is 11 times higher than the axial ones. In accordance with Figure 4 and Table 2, with the indication of statistical characteristics of the presented graphs, when changing the winding angle of the wrapping thread by 65° and 75°, the circumferential stresses from the prestressing decreased by 1.4 times and 1.2 times, respectively, and the axial stresses increased by five and three times, which is two and four times lower than the circumferential ones. The above effect in the wall is explained by the redistribution of forces due to the change in the winding angle of the wrapping thread.

**Figure 5:**
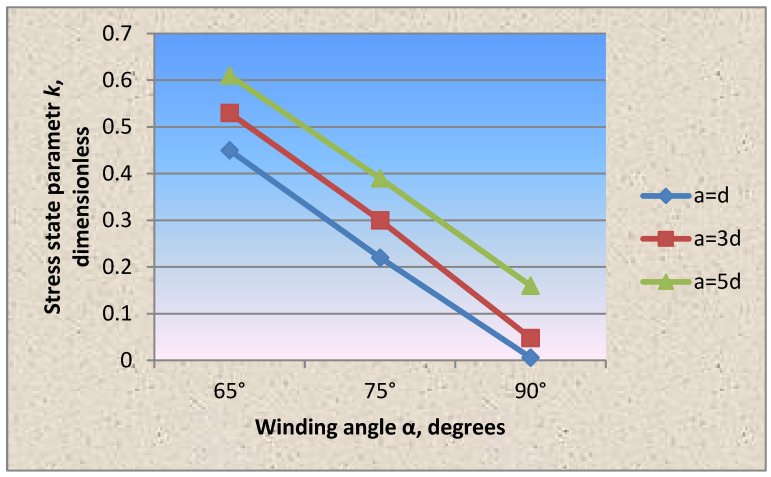
Dependence of the stress state parameter on the winding angle of the wrapping thread with the winding wire diameter of 1.0 mm.

The influence of the winding angle of the wrapping thread on the stress state parameter characterizing the strength balance of the wall (the ratio of circumferential stresses to axial stresses) is presented in the form of dependencies in accordance with Figure 5, from which it can be deduced that a decrease in the thread’s winding angle from 90° to 65° to the longitudinal shell axis favorably influences the stressed state of the shell wall and confirms the assertion that by changing the prestressing parameters, it is possible to control the stress state of the stressed state of structures and obtain an equally strong structure.

The same effect is observed in accordance with Figure 4 and Figure 5 in the cylindrical shell wall fragment with a wire wrapping with a diameter of 1.0 mm and a pitch of 3d, 5d.

As in the case of dense winding of the wire thread at an angle of 90°, the circumferential stresses in the shell wall under the wire exceeded the axial ones by 11 times. Changing the winding angle of the wrapping thread in the direction of decreasing to 65° and 75° reduced the circumferential stress values, respectively, by 1.4 and 1.2 times, and the axial stresses increased by 5 and 3 times. The stress state parameter, in accordance with Figure 5, when winding the thread at an angle of 90° = 0.12, 75° = 0.4 and 65° = 0.65.

In accordance with the tasks of experimental studies, in addition to measuring the stresses under the wrapping wire, the circumferential and axial stresses were measured at points between the wrapping turns at eight characteristic points of the shell fragment section. The circumferential stresses from the prestressing in the spans between the turns, depending on the winding pitch of the wrapping thread, when the winding angle was changed from 90° to 65°, decreased by 1.8 and 1.5 times, respectively, and the axial stresses increased by five and three times, respectively.

In general, note that with an increase in the wrapping thread’s winding pitch due to an increase in the stresses in the spans between the turns, the prestressing efficiency decreases.

**Figure 6:**
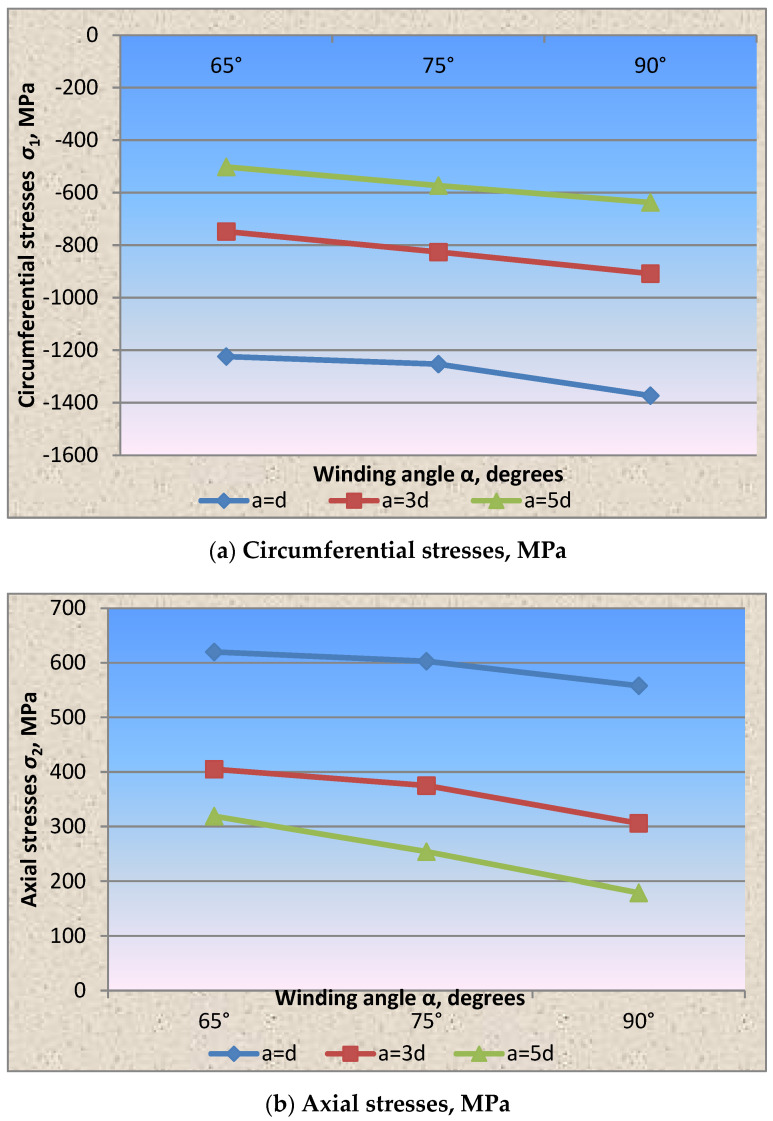
Dependence of the circumferential (**a**) and axial (**b**) stresses in the shell wall fragment on the winding angle with the winding wire diameter of 2.0 mm.

**Figure 7:**
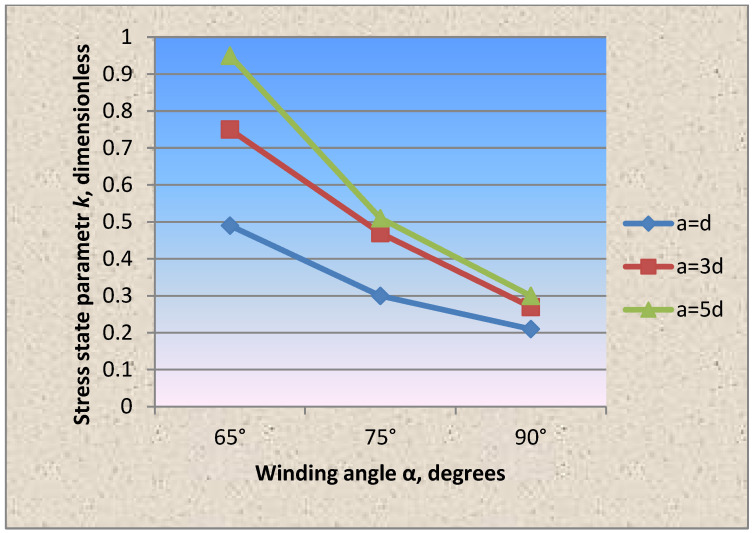
Dependence of the stress state parameter on the winding angle of the wrapping thread with the winding wire diameter of 2.0 mm.

**At the second stage** of experimental studies, the winding wire diameter was changed, which was taken equal to 2.0 mm (Figure 6 and Figure 7). The results of experimental studies are also shown in Table 2.

Comparison of the circumferential and axial stress values from the restressing in the shell wall fragment showed the same nature of stress distribution as in the case of winding wire with a diameter of 1.0 mm.

**Figure 8:**
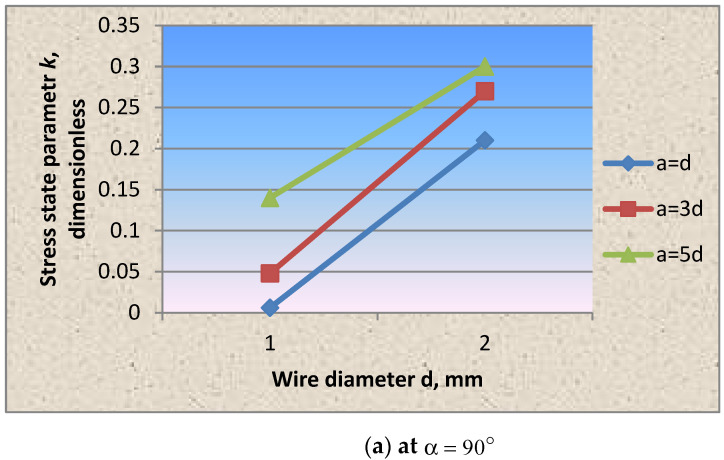

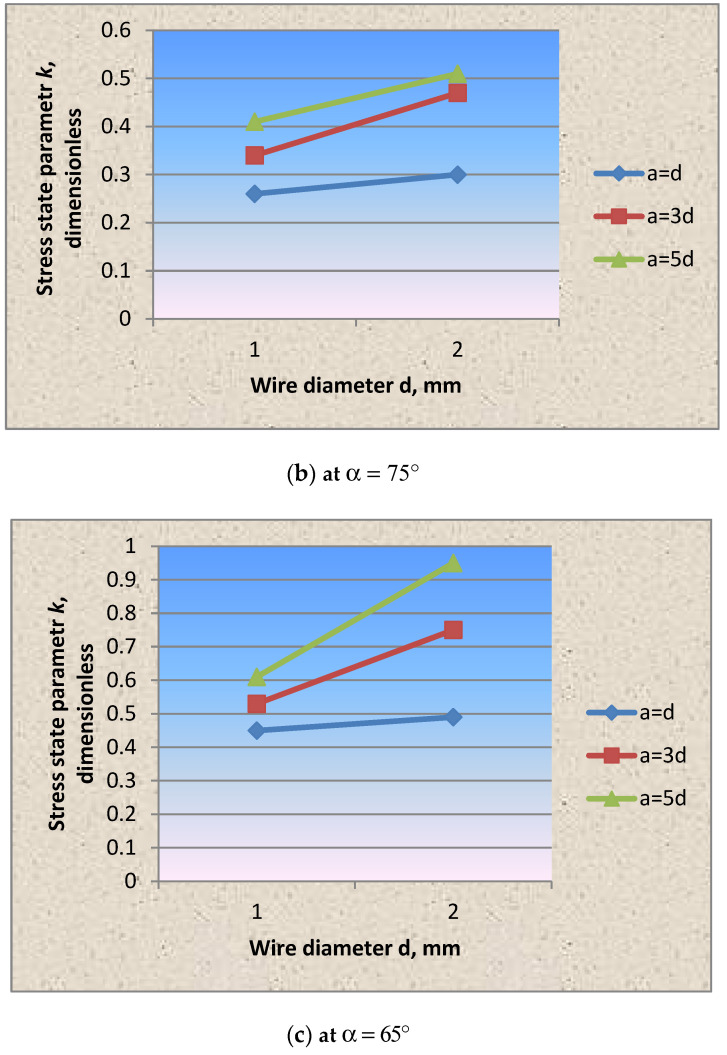
Dependence of the stress state parameter on the winding wire thickness at fixed values of the winding angle of the wrapping thread: (**a**) at a winding angle of 90 degrees, (**b**) at a winding angle of 75 degrees, (**c**) at a winding angle of 65 degrees.

However, it should be noted that, in comparison with the option of winding wire with a diameter of 1.0 mm, in this case, the stress state parameter values change (Figure 8).

It was found that together with the wrapping thread’s winding angle, the diameter of the wire selected for wrapping can be the structural control parameter that allows controlling the stressed state of the prestressed steel shell structure.

The research results confirmed the statement mentioned earlier in [22] that the winding thickness significantly affects the axial and circumferential stresses’ redistribution, and, consequently, the stress state parameter k. With an increase in the winding thickness, the parameter k, as in cases of wire winding without tension and with tension, increases, which means that the axial stress proportion also increases.

The analysis of experimental studies of a prestressed cylindrical shell is presented below for Stage-I and Sub-stage I-A, according to Table 1 and Table 2.

As a result of experimental studies of the prestressed shell fragment, the fact of an increase in the prestressing efficiency with an increase in the wrapping thread’s winding pitch was confirmed. However, increasing the stresses between the wrapping turns requires the search for their optimal parameters.

In any case, the pitch, according to the recommendations of [22,23,24,25,26,27,28,29,30,31,32,33,34,35,36,37,38,39,40], should be less than the half-wave length—$2.5\sqrt{r\delta }$, and it should be selected according to the conditions of the tasks solved by prestressing and a technical and economic comparison of options.

Tests of cylindrical shell fragments of various wall diameters and thicknesses once again confirmed the statement that the wrapping angle, pitch, tension force, and diameter are those parameters of the prestressed shell which can control the stress–strain state of the cylindrical shell’s strengthened wrapping and are selected in accordance with the tasks solved using the prestressing method.

The prestressed shell model wall’s stress–strain state was analyzed at various prestressing parameters.

The shell model fragments of various geometric parameters (shell diameter, shell wall thickness, steel wrapping diameter) as well as structural parameters (winding angle, winding pitch, winding tension forces) were studied, where the shell structure’s strength characteristics were estimated, which were achieved through the use of prestressing.

It was experimentally established that the circumferential and axial stresses in the shell wall fragment showed that the greatest circumferential compressive stresses in the shell wall due to prestressing occur when the winding is perpendicular to the longitudinal axis, which is 11 times higher than the axial ones. In accordance with Figure 4, when changing the winding angle of the wrapping thread by 65° and 75°, the circumferential stresses from the prestressing decreased by 1.4 times and 1.2 times, respectively, and the axial stresses increased by five and three times, which is two and four times lower than the circumferential ones. The above effect in the wall is explained by the redistribution of forces due to the change in the wrapping thread’s winding angle.

It is worth noting that such a nature of the stress distribution was shown both in the case of winding the wire with a diameter of 1.0 mm and with a diameter of 2.0 mm.

However, it should be noted that in comparison with the option of winding the wire with a diameter of 1.0 mm, when winding the wire with a diameter of 2.0 mm, the stress state parameter values change (Figure 8).

Using the ability to control the prestressed shell’s stress–strain state by changing significantly, mainly structural parameters, it is possible to more fully use the bearing capacity. This allows creating a high-tech design that is optimal for these operating conditions, which can positively complement the studies previously carried out in this direction.

In this case, prestressing is applied either to increase the bearing capacity of the operated shell or to reduce the wall thickness of the newly designed shell. In the latter case, the thick wall is replaced by a relatively thin, prestressed, high-strength wire wrapped around it. As a result, the structure is facilitated, metal is saved, the technology is simplified and the cost of structures is reduced. The results of the research confirm the possibility of using prestress taking into account the parameters for different cases in a cylindrical shell [22,23,24,25,26,27,28,29,30,31,32,33,34,35,36,37,38,39,40,41,42,43,44,45,46,47,48,49].

It is worth noting that the friction between the shell body and the wrapping thread was not taken into account in experimental studies [50,51,52].

There were also difficulties in experimentally determining the force in the wrapping wire during the test. However, these assumptions do not significantly affect the possibility of evaluating the features of the work of prestressed structures, and they can be taken into account during additional tests.

The main qualitative features of the processes occurring in the prestressed shell, due to the relative simplicity of the research model, were carried out only with respect to straight sections of above-ground shells. This allowed testing the scientific idea, substantiating some assumptions and select structural solutions. In this regard, as a main shortcomings and limitations of the study, it can be noted that in the future, it is necessary to expand the field of study of prestressed shells in the direction of studying curved sections of shell structures, in the direction of using composite materials as a winding and a comprehensive system analysis, which will also take into account the friction forces between the winding and the shell sink.

## 4. Conclusions

The modeling method chosen in the work, based on the classical theory of mechanical similarity and modeling, allowed satisfactorily transferring the results of testing the cylindrical shell fragments to real structures.

The applied research method and measuring equipment allowed fulfilling the goal set for experimental studies in full.

It was found that when the winding angle of the wrapping thread is changed from perpendicular to the shell axis to 75° and 65°, the circumferential stresses decrease, and the axial stresses increase (Figure 4).

It was found that the change in the wrapping thread’s winding angle results in the stress redistribution in the shell wall and favorably affects the wall’s stress state parameter, i.e., the structural strength balance.

With an increase in the winding wire diameter from 1 to 2 mm and a change in the winding angle, the nature of the stress distribution and the stress state parameter does not change.

The cylindrical shell fragments’ tests confirmed the fact of increasing the efficiency of prestressing with the increase in the winding pitch of the wrapping thread. However, it is indicated that its optimal value depends on the stresses arising between the wrapping turns.

The cylindrical shell fragments’ experimental studies confirmed the assertion that by changing the structural parameters of prestressing, it is possible to control the stress state of the stressed state of structures and obtain an equally strong structure.

Based on testing the cylindrical shell fragments, the stress state parameter dependencies on the winding wire’s wrapping angle, pitch and thickness were experimentally obtained for the first time (Figure 4, Figure 5, Figure 6, Figure 7 and Figure 8).

## Figures and Tables

**Table 1:** Stages, sub-stages and series of experiments.

Stages	Sub-Stages	Series	Number of Fragments, Unit	Diameter/Thickness of Full-Scale Shell, mm	Modeling Method	Shell Fragment Diameter, mm	Shell Fragment Wall Thickness, mm	Winding Wire Diameter, mm	Wire Pitch	Winding Angle, Degrees
1	2	3	4	5	6	7	8	9	10	11
I	I-A	I-A-1/1 *	2	720/5.0	affine	360	0.5	1.0/2.0	d	90
		I-A-2/2 *	2	720/5.0	affine	360	0.5	1.0/2.0	3d	90
		I-A-3/3 *	2	720/5.0	affine	360	0.5	1.0/2.0	5d	90
		I-A-4/4 *	2	720/5.0	affine	360	0.5	1.0/2.0	d	75
		I-A-5/5 *	2	720/5.0	affine	360	0.5	1.0/2.0	3d	75
		I-A-6/6 *	2	720/5.0	affine	360	0.5	1.0/2.0	5d	75
		I-A-7/7 *	2	720/5.0	affine	360	0.5	1.0/2.0	d	65
		I-A-8/8 *	2	720/5.0	affine	360	0.5	1.0/2.0	3d	65
		I-A-9/9 *	2	720/5.0	affine	360	0.5	1.0/2.0	5d	65
	I-B	I-B-1/1 *	2	720/6.0	affine	360	0.6	1.0/2.0	d	90
		I-B-2/2 *	2	720/6.0	affine	360	0.6	1.0/2.0	3d	90
		I-B-3/3 *	2	720/6.0	affine	360	0.6	1.0/2.0	5d	90
		I-B-4/4 *	2	720/6.0	affine	360	0.6	1.0/2.0	d	75
		I-B-5/5 *	2	720/6.0	affine	360	0.6	1.0/2.0	3d	75
		I-B-6/6 *	2	720/6.0	affine	360	0.6	1.0/2.0	5d	75
		I-B-7/7 *	2	720/6.0	affine	360	0.6	1.0/2.0	d	65
		I-B-8/8 *	2	720/6.0	affine	360	0.6	1.0/2.0	3d	65
		I-B-9/9 *	2	720/6.0	affine	360	0.6	1.0/2.0	5d	65
II	II-A	II-A-1/1 *	2	1020/6.0	affine	510	0.6	1.0/2.0	d	90
		II-A-2/2 *	2	1020/6.0	affine	510	0.6	1.0/2.0	3d	90
		IIA-3/3 *	2	1020/6.0	affine	510	0.6	1.0/2.0	5d	90
		II-A-4/4 *	2	1020/6.0	affine	510	0.6	1.0/2.0	d	75
		II-A-5/5 *	2	1020/6.0	affine	510	0.6	1.0/2.0	3d	75
		II-A-6/6 *	2	1020/6.0	affine	510	0.6	1.0/2.0	5d	75
		II-A-7/7 *	2	1020/6.0	affine	510	0.6	1.0/2.0	d	65
		II-A-8/8 *	2	1020/6.0	affine	510	0.6	1.0/2.0	3d	65
		II-A-9/9 *	2	1020/6.0	affine	510	0.6	1.0/2.0	5d	65
	II-B	II-B-1/1 *	2	1020/7.0	affine	510	0.7	1.0/2.0	d	90
		II-B-2/2 *	2	1020/7.0	affine	510	0.7	1.0/2.0	3d	90
		II-B-3/3 *	2	1020/7.0	affine	510	0.7	1.0/2.0	5d	90
		II-B-4/4 *	2	1020/7.0	affine	510	0.7	1.0/2.0	d	75
		II-B-5/5 *	2	1020/7.0	affine	510	0.7	1.0/2.0	3d	75
		II-B-6/6 *	2	1020/7.0	affine	510	0.7	1.0/2.0	5d	75
		II-B-7/7 *	2	1020/7.0	affine	510	0.7	1.0/2.0	d	65
		II-B-8/8 *	2	1020/7.0	affine	510	0.7	1.0/2.0	3d	65
		II-B-9/9 *	2	1020/7.0	affine	510	0.7	1.0/2.0	5d	65
III	III-A	II-A-1/1 *	2	1220/5.0	direct	122	0.5	1.0/2.0	d	90
		II-A-2/2 *	2	1220/5.0	direct	122	0.5	1.0/2.0	3d	90
		IIA-3/3 *	2	1220/5.0	direct	122	0.5	1.0/2.0	5d	90
		II-A-4/4 *	2	1220/5.0	direct	122	0.5	1.0/2.0	d	75
		II-A-5/5 *	2	1220/5.0	direct	122	0.5	1.0/2.0	3d	75
		II-A-6/6 *	2	1220/5.0	direct	122	0.5	1.0/2.0	5d	75
		II-A-7/7 *	2	1220/5.0	direct	122	0.5	1.0/2.0	d	65
		II-A-8/8 *	2	1220/5.0	direct	122	0.5	1.0/2.0	3d	65
		II-A-9/9 *	2	1020/5.0	direct	122	0.5	1.0/2.0	5d	65

Note: Without an asterisk is the winding thickness of 1 mm, with an asterisk is the winding thickness of 2 mm.

**Table 2:** Experimental data on the axial and circumferential stresses of the prestressed shell fragment.

Series	Winding Angle, α	Experimental Values			Series	Winding Angle, α	Experimental Values		
		$\sigma_{1c}\;(\mathrm{kN}/{\mathrm{cm}}^{2})$	$\sigma_{2c}\;(\mathrm{kN}/{\mathrm{cm}}^{2})$	k			$\sigma_{1c}\;(\mathrm{kN}/{\mathrm{cm}}^{2})$	$\sigma_{2c}\;(\mathrm{kN}/{\mathrm{cm}}^{2})$	k
		Average	Average				Average	Average	
I-A-1	90	−1948	167	0.006	II-A-5 *	75	−849	308	0.36
I-A-1 *	90	−1373	319	0.21	II-A-6	75	−627	217	0.35
I-A-2	90	−1074	150	0.041	II-A-6 *	75	−648	0383	0.60
I-A-2 *	90	−908	254	0.27	II-A-7	65	−1658	802	0.48
I-A-3	90	−841	98	0.14	II-A-7 *	65	1204	644	0.54
I-A-3 *	90	−637	179	0.30	II-A-8	65	−902	432	0.48
I-A-4	75	−1921	504	0.26	II-A-8 *	65	−721	458	0.63
I-A-4 *	75	−1253	386	0.3	II-A-9	65	−597	333	0.56
I-A-5	75	−1031	317	0.34	II-A-9 *	65	−531	506	0.94
I-A-5 *	75	−826	391	0.47	II-B-1	90	−1812	3.1	0.003
I-A-6	75	−750	310	0.41	II-B-1 *	90	−1300	13.4	0.01
I-A-6 *	75	−573	306	0,51	II-B-2	90	−887	20.8	0.023
I-A-7	65	−1796	817	0.45	II-B-2 *	90	−776	76	0.09
I-A-7 *	65	−1224	602	0.49	II-B-3	90	−678	40	0.06
I-A-8	65	−955	506	0.53	II-B-3 *	90	−511	154	0.3
I-A-8 *	65	−748	603	0.75	II-B-4	75	−1712	480	0.28
I-A-9	65	−676	413	0.61	II-B-4 *	75	−1210	394	0.32
I-A-9 *	65	−502	558	0.95	II-B-5	75	−809	245	0.3
I-B-1	90	−1943	11.5	0.005	II-B-5 *	75	−711	333	0.47
I-B-1 *	90	−1348	26	0.019	II-B-6	75	−510	227	0.44
I-B-2	90	−1163	44	0.039	II-B-6 *	75	−475	335	0.7
I-B-2 *	90	−845	142	0.16	II-B-7	65	−1566	717	0.45
I-B-3	90	−687	75	0.1	II-B-7 *	65	−1092	634	0.58
I-B-3 *	90	−565	313	0.55	II-B-8	65	−748	431	0.57
I-B-4	75	−1823	511	0.28	II-B-8 *	65	−665	405	0.6
I-B-4 *	75	−1180	426	0.36	II-B-9	65	−493	284	0.58
I-B-5	75	−929	300	0.32	II-B-9 *	65	−434	432	0.99
I-B-5 *	75	−857	319	0.38	III-A-1	90	1994	31	0.015
I-B-6	75	−634	252	0.39	III-A-1 *	90	−1352	87	0.06
I-B-6 *	75	−610	490	0.81	III-A-2	90	−1354	179	0.13
I-B-7	65	−1710	823	0.48	III-A-2 *	90	−707	403	0.57
I-B-7 *	65	−1197	591	0.49	III-A-3	90	−384	302	0.44
I-B-8	65	−1008	499	0.49	III-A-3 *	90	−650	531	0.81
I-B-8 *	65	−771	535	0.64	III-A-4	75	−1853	512	0.3
I-B-9	65	−589	366	0.62	III-A-4 *	75	−1296	433	0.33
I-B-9 *	65	−491	632	1.21	III-A-5	75	−1003	445	0.44
II-A-1	90	−1841	6.9	0.004	III-A-5 *	75	−630	609	0.96
II-A-1 *	90	−1573	17.5	0.01	III-A-6	75	−650	507	0.78
II-A-2	90	−960	28	0.028	III-A-6 *	75	−608	779	1.28
II-A-2 *	90	−809	118	0.14	III-A-7	65	−1823	654	0.36
II-A-3	90	−627	52	0.08	III-A-7 *	65	−1183	684	0.57
II-A-3 *	90	−695	217	0.31	III-A-8	65	−945	643	0.68
II-A-4	75	−1746	489	0.28	III-A-8 *	65	−574	724	1.26
II-A-4 *	75	1307	377	0.29	III-A-9	65	−602	632	1.04
II-A-5	75	−919	285	0.30	III-A-9 *	65	598	875	1.46

*: is the winding thickness of 2 mm.

## Data Availability

Data sharing is not applicable to this article.
